# Scanning MRI condtional implantable devices

**DOI:** 10.1186/1532-429X-16-S1-T10

**Published:** 2014-01-16

**Authors:** Michelle Walkden, Alison M Fletcher, Charles Peebles, James Shambrook, Stephen Harden

**Affiliations:** 1 Cardiothoracic Radiology, University Hospital Southampton, Southampton, Hampshire, UK

## Background

New developments and expanding indications have lead to a significant increase in the number of pacemakers and loop recorders being implanted. Alongside this cardiac magnetic resonance imaging (CMRI) is increasing being utilised in the assessment of cardiac disease. In the UK, MRI conditional devices are often now being implanted as the device of choice by cardiologists as it is assumed the patient may need an MRI scan at some stage in their lifetime. The number of patients that present to the MRI scanner with a cardiac device is likely to increase rapidly.

## Methods

Implantable cardiac devices can produce significant image degradation for cardiac MRI and steady state free precession (SSFP) sequences are particularly affected. This is primarily due to inhomogenieties within the magnet field caused by the pacemaker generator or loop recorder. To overcome these image artefacts it is necessary to alter the pulse sequence we use, switching to a gradient echo sequence which produces less image artefact. Although these sequences have a lower image contrast the images obtained are of more diagnostic quality. In our experience fast spin echo sequences appear virtually unaffected with only minimal artefact directly adjacent to the device. Phase contrast imaging demonstrates artefact but the images remain diagnostic. For reproducibility, the majority of cardiac MRI patients are scanned on arrested expiration, however in patients with implanted cardiac devices it can be advantageous to scan on arrested inspiration including SSFP imaging. This has the effect of increasing the distance between the heart and the device which helps minimise image artefact over both ventricles.

## Results

We have found that loop recorders produce significantly more image artefact which can be problematic for ventricular function assessment, even when a GRE sequence is used. Many of the patients with these devices have reported experiencing slight heating and tugging of the device despite staff following the manufacturers guidelines when scanning. We have found that it is not always necessary to switch to GRE sequences with MR conditional pacemakers due to the artefact not always impeding ventricular wall motion assessment. Lead related artefacts are minimal. From the patients we have scanned so far none have reported any effects similar to those with loop recorders.

## Conclusions

A full range of CMR sequences including perfusion imaging are used in our department. From our experience of scanning implantable cardiac devices employing these techniques ensures that we can achieve the highest quality diagnostic images in these patients.

## Funding

None.

